# Scaffold-Mediated Gene Delivery for Osteochondral Repair

**DOI:** 10.3390/pharmaceutics12100930

**Published:** 2020-09-29

**Authors:** Henning Madry, Jagadeesh Kumar Venkatesan, Natalia Carballo-Pedrares, Ana Rey-Rico, Magali Cucchiarini

**Affiliations:** 1 Center of Experimental Orthopaedics, Saarland University Medical Center, Kirrbergerstr. Bldg 37, D-66421 Homburg, Germany; henning.madry@uks.eu (H.M.); jegadish.venki@gmail.com (J.K.V.); 2 Cell Therapy and Regenerative Medicine Unit, Centro de Investigacións Científicas Avanzadas (CICA), Universidade da Coruña, S-15071 A Coruña, Spain; natalia.carballo@udc.es (N.C.-P.); ana.rey.rico@udc.es (A.R.-R.)

**Keywords:** osteochondral repair, gene therapy, tissue engineering, controlled delivery

## Abstract

Osteochondral defects involve both the articular cartilage and the underlying subchondral bone. If left untreated, they may lead to osteoarthritis. Advanced biomaterial-guided delivery of gene vectors has recently emerged as an attractive therapeutic concept for osteochondral repair. The goal of this review is to provide an overview of the variety of biomaterials employed as nonviral or viral gene carriers for osteochondral repair approaches both in vitro and in vivo, including hydrogels, solid scaffolds, and hybrid materials. The data show that a site-specific delivery of therapeutic gene vectors in the context of acellular or cellular strategies allows for a spatial and temporal control of osteochondral neotissue composition in vitro. In vivo, implantation of acellular hydrogels loaded with nonviral or viral vectors has been reported to significantly improve osteochondral repair in translational defect models. These advances support the concept of scaffold-mediated gene delivery for osteochondral repair.

## 1. Introduction

**Figure 1:**
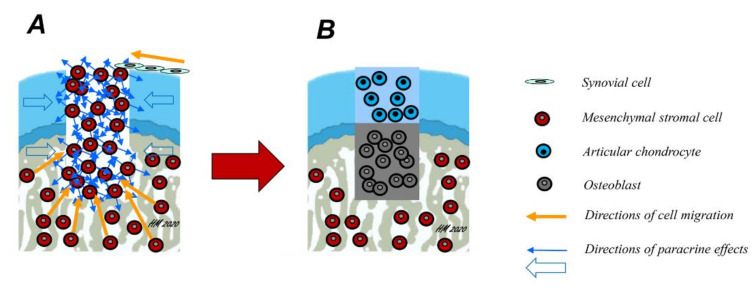
Mechanisms of osteochondral repair. Osteochondral defects involve, by definition, both the articular cartilage and the subchondral bone. Spontaneously, they are mainly repaired by mesenchymal stromal cells (MSCs) arising from the subchondral bone marrow (**A**, yellow arrows). However, some cell migration into the defect from synovial cells is also possible. Over time, these cells differentiate either into chondrocytes or osteoblasts and deposit their extracellular matrix (ECM), depending on their location within the osteochondral defect, a mechanism possibly regulated in part by paracrine effects of the cells in the adjacent osteochondral unit (blue arrows). Ideally, a fibrocartilaginous repair tissue forms in the upper part of the defect (**B**), while the subchondral bone is repaired with new bone.

Articular cartilage, the gliding tissue covering the ends of all joints, has a reduced ability for repair [1]. Osteochondral defects are areas of joint damage that involve both the articular cartilage and the underlying subchondral bone (Figure 1). Such defects often result from an acute traumatic injury to the joint or are caused by an underlying disorder of the subchondral bone, for example osteochondritis dissecans (OCD) that secondarily affects the cartilage.

Surgical repair techniques for osteochondral defects focus on simultaneously restoring the subchondral bone and a cartilaginous repair tissue [2]. While the quality of (osteo)chondral repair is often regarded as a sole outcome and criterion for success, translational evidence shows that even small lesions can be the starting point of osteoarthritis (OA) development in the vicinity of the defect [3]. OA originating from such defects may encroach on formerly unaffected areas of the affected compartment and progressively involve the entire joint [3]. Such OA is challenging as it may be present long before arising to a clinically symptomatic state [4]. Long-term clinical evaluations attest to the high rate of OA in the case of untreated large osteochondral defects such as those occurring in OCD [5]. Surgical restoration of the osteochondral unit leads to good long-term outcomes in such cases [6,7]. However, many patients suffering from advanced OA may require total joint arthroplasty, a surgical end-stage treatment using implants that over time may pose problems such as loosening or infection. To avoid arthroplasty, especially in younger patients, much effort has been dedicated to the treatment of osteochondral defects at early stages to provide for long-term repair and prevent the development and progression of secondary OA.

Emerging treatments include cell-based and acellular, scaffold-based tissue engineering and gene therapy. The standard cell-based therapy to repair cartilage defects is the autologous chondrocyte implantation (ACI), with good long-term outcomes [8]. Mesenchymal stromal/stem cells (MSCs) from the bone marrow or from connective tissues like fat are being pursued as alternatives for cartilage repair and are applied via intra-articular administration in patients with knee OA. Early-phase clinical studies provide some promising insight into their efficacy, but the mechanisms of action involved remains unclear [9].

A potential advantage of gene therapy is the local delivery of gene sequences coding for therapeutic factors with a known ability to promote both cartilage and bone reparative processes. Herein, multiple growth factors have been identified as potent mediators to promote chondrogenesis, osteogenesis, and/or angiogenesis [10,11,12]. In recent years, the successful genetic modification of cells of the musculoskeletal system, among which articular chondrocytes and MSCs, either using viral or nonviral vectors, has been achieved to efficiently deliver therapeutic genes, enhancing their regenerative capacities [13,14]. Moreover, different scaffolds have been used to support the delivery of recombinant genes and gene combinations via gene transfer using both nonviral and viral vectors to target cells relevant of osteochondral tissue engineering and repair in vitro and in vivo. The development of such bioactive osteochondral implants that circumvent the need for ex vivo tissue generation allows for an in situ tissue engineering based on the active transgene product in vivo. The goal of the present article is to provide an overview of the current advances in scaffold-mediated gene delivery for osteochondral repair.

## 2. Candidate Genes for Osteochondral Repair

**Figure 2:**
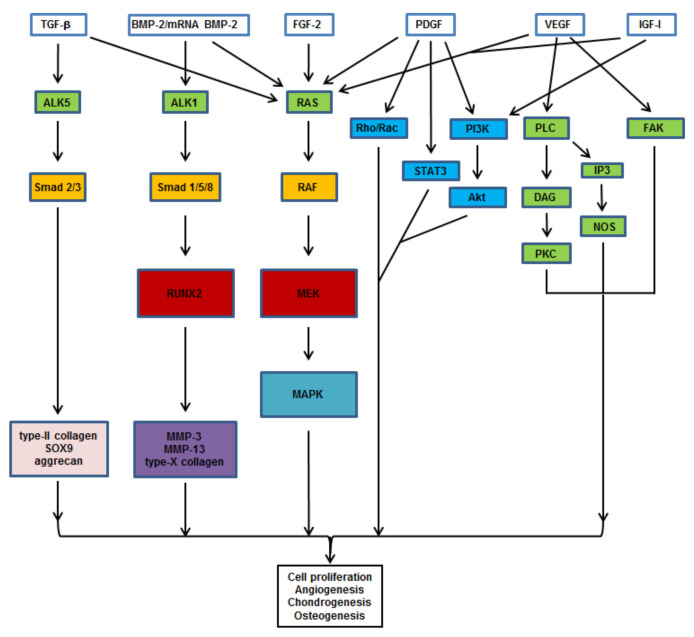
Signaling pathways triggered by therapeutic candidates for osteochondral repair. TGF-β promotes chondrogenesis by activating the PI3K, Smad 2/3, and RhoA pathways. FGF-2, BMP-2, and mRNA BMP-2 induce osteo-/chondrogenesis via the RAS/RAF/MEK/MAPK and Smad pathways. PDGF activates angiogenesis via crosstalks between the MAPK, Rho/Rac, STAT3, and PI3K pathways. IGF-I induces the MAPK and PI3K pathways. VEGF induces angiogenesis by activating the PLC, IP3, and FAK pathways. Abbreviations: TGF-β: transforming growth factor beta; BMP-2: bone morphogenetic protein 2; mRNA: messenger ribonucleic acid; FGF-2: basic fibroblast growth factor; PDGF: platelet-derived growth factor; VEGF: vascular endothelial growth factor; IGF-I: insulin-like growth factor I; ALK: activin receptor-like kinase; RAS: Rat sarcoma; RhoA: Ras homolog gene family, member A; Rac: Ras-related C3 botulinum toxin substrate; PI3K: phosphatidylinositol-4,5-bisphosphate 3-kinase; PLC: phosphoinositide phospholipase C; FAK: focal adhesion kinase; STAT3: signal transducer and activator of transcription 3; IP3: inositol 1,4,5-trisphosphate; Smad: suppressor of mothers against decapentaplegic; RAF: rapidly accelerated fibrosarcoma; Akt/PKB: protein kinase B; DAG: diacylglycerol; NOS: nitric oxide synthase; PKC: protein kinase C; RUNX2: Cbfa-1/runt-related transcription factor 2; MEK: mitogen-activated protein kinase kinase; MAPK: MAPK: mitogen-activated protein kinase; SOX9: sex determining region Y-box 9; MMP: matrix metalloproteinase.

Polypeptide growth factors including the transforming growth factor beta (TGF-β) [15,16,17], the insulin-like growth factor I (IGF-I) [18,19], and the basic fibroblast growth factor (FGF-2) [20,21] play important roles in bone and cartilage repair by modulating ossification and enhancing the expression of cartilage major ECM components (type-II collagen, aggrecan). Due to their potent osteogenic effects, the members from the bone morphogenetic protein (BMP) superfamily, and particularly BMP-2, have been applied to trigger osteogenesis and mineralization leading to the expression of bone-related markers (osteopontin, osteocalcin, alkaline phosphatase) [22]. The BMP-2 isoform [15], as well as BMP-7 [23], also plays important roles in chondrogenesis by stimulating cell differentiation and the production of the cartilage ECM. In addition, angiogenic factors such as the vascular endothelial growth factor (VEGF) [24,25] and the platelet-derived growth factor (PDGF) [26,27] have also been described to promote successful bone and cartilage healing via the induction of osteochondral progenitors proliferation and proteoglycan deposition. Transcription factors as the cartilage-specific sex determining region Y-boxes (SOX) 5, 6, and 9 (SOX5, SOX6, and SOX9, or SOX trio) [28] are also potential candidates for osteochondral repair. These factors are critically involved in the formation and maintenance of the cartilage by activating the expression of major matrix components. Other factors include the bone-specific Cbfa-1/runt-related transcription factor 2 (RUNX2) [29] that modulates osteoblast differentiation with endochondral and membranous ossification, and osterix (OSX) that is required for bone maintenance in synergism with RUNX2 [30]. More recently, the use of messenger ribonucleic acids (mRNAs) for these various factors has been evoked as tools for therapy, being potentially amenable to scaffold-mediated delivery in particular for strategies that aim at initiating osteochondral repair [31,32,33,34]. Figure 2 presents an overview of the pathways targeted by these various candidates.

## 3. Nonviral Gene Delivery Systems

Gene transfer via nonviral vectors (transfection) is the incorporation of the DNA plasmid (pDNA) either alone or complexed with cationic or ionizable lipids (lipoplexes), cationic polymers (polyplexes), or a combination of both (lipopolyplexes) [35] into the target cell population [36] (Table 1). More recent approaches also include the use of niosomes (nioplexes) [37], dendrimers (dendriplexes) [38], and gold or carbon nanostructures [39]. Nonviral vectors are generally considered safe carriers compared with viral constructs as they do not carry the risk of insertional mutagenesis (nonviral vectors are kept under episomal forms) and have a low immunogenicity (nonviral vectors have no intrinsic viral coding sequences) [40]. However, nonviral gene transfer is characterized by a comparable low transfection efficiency, limiting the production of high amounts of the therapeutic protein. Extensive research has been performed during the last decades by identifying optimal promoters and designing new vectors in order to improve their performance. Various promoters, including the human cytomegalovirus immediate-early (CMV-IE), simian vacuolating virus 40 (SV40), and elongation factor (EF)-1 have been tested to achieve high levels of protein expression in different cell lines and primary cell cultures [41,42,43]. Likewise, different pDNA conformations namely mini pDNA [44] and DNA mini-strings [45] based on DNA-mini linear covalently closed DNA vectors have been designed, exhibiting better cytoplasmic kinetics and improved transfection efficiently compared with parental plasmids. Alternative approaches include the use of integrative nonviral transposon systems as those based on *Sleeping Beauty* [46] or *PiggyBac* transposons [47]. These systems rely on the simultaneous delivery of two pDNA one containing the gene of interest flanked by the transposase recognizable terminal inverted repeats (TIRs) and another pDNAs containing the transposase gene. Due to its integration capacity and nonviral nature, transposons constitute a safer alternative to the use of viral vectors in different gene therapy protocols [48].

## 4. Viral Vectors

Recombinant viral vectors are divided into different groups according to the original type of virus they are based upon: adenovirus, retrovirus, baculovirus, and adeno-associated virus (AAV) [49] (Table 1).

### 4.1. Adenoviral Vectors

Among the viral systems employed for gene therapy, adenoviruses have been used often because of their high transduction efficiencies and transgene expression in various types of cells, potentially important for in vivo approaches. More than 50 adenovirus serotypes are available for gene therapy approaches. Adenovirus serotype 5 (Ad5) has been briefly used in both in vitro and in vivo studies. Adenoviral vectors have been used to transfer growth factor genes (TGF-β, FGF-2, IGF-I, BMPs, and the growth and differentiation factor 5—GDF-5) into cells of the musculoskeletal system [50,51,52,53,54,55,56,57,58,59]. Direct delivery via adenoviral-mediated transduction of IGF-I in synovial tissue in the metacarpophalangeal joints of horses [60] and of BMP-2 injected directly in osteochondral defects in vivo [61], or together with a decalcified cortical bone matrix as scaffold containing the vector particles has been achieved [62]. However, the major challenges limiting the success of adenoviral approaches are the considerable immune responses [63] particularly with a view towards a clinical applications.

### 4.2. Retroviral Vectors

Retroviruses have the advantage of integrating their DNA into the host genome, allowing them to maintain gene expression for longer periods of time [64]. In comparison to the adenoviral vectors, fewer studies employed retroviral vectors for the delivery of growth factors, such as TGF-β, SOXs, BMPs, or VEGF inhibitors, both in vitro and in vivo [65,66,67,68]. The main problem of this kind of vector is the risk of insertional mutagenesis and the potential activation of oncogenes. In addition, retroviral vectors transduce only dividing cells with a restricted host range and low efficacy.

### 4.3. Baculoviral Vectors

Baculoviruses show no pathogenicity toward humans and can be used under biosafety level 2 conditions. Baculoviral vectors, like adenoviral vectors, have been shown to transduce both dividing and non-dividing mammalian cells, including articular chondrocytes and adipose-derived MSCs with TGF-β [69] and BMPs [70] in vitro and in vivo. However, baculoviral vectors are not able to replicate and do not integrate their DNA into the chromosomes of transduced mammalian cells, resulting in a transient transgene expression of less than 1 week. For these reasons, baculoviral vectors have attracted less research interests and their clinical application is not permitted.

### 4.4. Recombinant Adeno-Associated Viral (rAAV) Vectors

AAV is a small, non-pathogenic human parvovirus that is defective for replication. It has been genetically manipulated to form recombinant particles that lack all viral sequences and contain instead a transgene cassette. This feature therefore makes rAAV much less immunogenic than adenoviral gene vehicles that are not fully devoid of viral coding sequences. AAV are generally kept as stable episomes in the target cells, which allows them to support the long-term expression of the transgenes they carry (months to years). This characteristic further prevents the activation of oncogenes upon insertional mutagenesis as noted when using integrative retroviral vectors. rAAV target dividing and also non-dividing cells both at very high efficiencies, which enables direct gene transfer protocols in vivo. For cells that remain refractory to rAAV gene transfer, research has been developed to replace conventional rAAV vectors by pseudotyped, chimeric, hybrid, and self-complementary (scAAV) constructs to overcome the slow viral genome conversion from single-stranded to double-stranded DNA. Finally, the relatively limited gene delivery ability of rAAV (~4.7 kb) has been tackled by using the aptitude of the virus to form concatemers. Therefore, rAAV became a preferred gene transfer system for cartilage and osteochondral repair in vivo [21,53,71,72,73].

## 5. Scaffolds for Osteochondral Repair

The ideal scaffold for osteochondral repair is biocompatible, biodegradable and provides a three-dimensional (3D) environment, mimicking structural and biological cues of the native osteochondral unit, aiming to support both cartilaginous and subchondral bone repair in a bioinspired spatio-temporal fashion. Due to the differences in the mechanical properties and biological structure of the articular cartilage and the subchondral bone, the design of scaffolds for osteochondral repair needs to fulfill the requirements of both tissues [74]. In this scenario, the development of composite scaffolds is centered on the design of biomaterials with adequate mechanical properties to support subchondral bone restoration, while maintaining a comparably weaker structure allowing for cartilaginous repair [75,76]. Such composites may comprise bilayer and multilayer scaffolds, and also continuous gradient scaffolds [77].

Initially, the scaffold should provide a biomechanically strong support, with an adapted porous structure to permit cellular activities, together with an appropriate in vivo degradation rate that is in parallel to the ECM deposition. Combination of biomaterials with advanced technologies [78,79,80,81,82,83] has allowed for developing a new generation of 3D scaffolds with adapted features for cartilage and bone repair [84]. The technique of 3D printing has been also applied to generate osteochondral constructs that may be tailored in the future to match the often irregular osteochondral defects. A recent study engineered biphasic osteochondral constructs from 3D-printed fiber networks that mechanically reinforces alginate hydrogels whilst simultaneously supporting MSC chondrogenesis [83].

A variety of biomaterials have been employed in osteochondral tissue engineering, including hydrogels [34,82], solid scaffolds [85], and hybrid scaffolds [76]. Each are made of either natural cell-compatible materials or of synthetic compounds with more controllable properties as mono- or multiphasic systems (Table 2). While cells or gene vectors can be encapsulated in the 3D hydrogels, they are usually attached to the porous structures of solid scaffolds. Hydrogels are well adapted for cartilage repair as they have high water contents mimicking cartilage-based ECM glycosaminoglycans [86] and biocompatibility, often with lower mechanical properties compared with solid scaffolds [87,88]. Natural polymer biomaterials such as collagen [89], alginate [90], gellan gum [91], or silk fibroin [92] have been studied as scaffolds for cartilage repair due to their biocompatible nature promoting proliferation and differentiation of the encapsulated cells which makes them promising systems in various tissue engineering approaches [74,92,93]. Solid scaffolds are highly porous structures, allowing for migration and infiltration of cells from the surrounding tissue. They may originate from natural polymers such as poly-glycolic acid (PGA), poly (L, D-lactic-co-glycolic acid) (PLGA), polycaprolactone (PCL), polyurethane (PU), and polyethylene terephthalate. Synthetic biomaterials synthetic as PGA and their poly(lactide-co-glycolide) copolymers (PLGA) exhibit more reproducible physical and chemical properties but their degradation by-products may be toxic [94]. Inorganic materials like hydroxyapatite (HAp) have been mostly used in bone regeneration approaches, due to its exceptional mechanical stiffness and osteoinductivity [74,76]. Metallic scaffolds based on tantalum or titanium are also used as subchondral bone substitutes alone or in combination with other biomaterials in composite scaffolds [95,96,97]. Their lack of degradation and the possibility of releasing corrosion products are concerns to be considered [74]. Finally, a variety of hybrid scaffolds based on solid scaffolds and hydrogels has been prepared, for example by combining fibrin hydrogels with solid PU scaffolds.

Noteworthily, biomaterial scaffolds may have a significant impact on immune responses and foreign body reactions due to their physical, chemical, and biological properties. Herein, both the form of biomaterial (hydrogel, solid matrix, or micro/nanoparticles), degradation profile, level of crosslinking, hydrophobicity, topography, ad biomaterial origin (natural versus synthetic) are important parameters to consider when designing an ideal scaffold for osteochondral repair [98].

**Figure 3:**
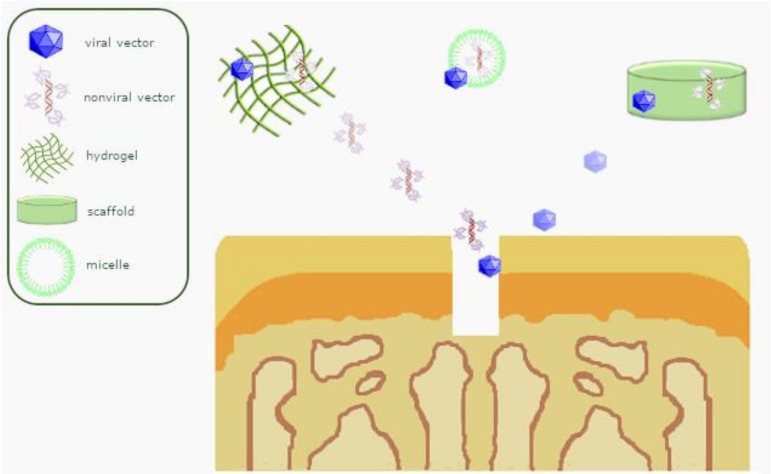
Overview of scaffold-guided gene transfer mechanisms for osteochondral repair.

Scaffold-guided gene transfer for the goal of cartilage repair (Figure 3) has been attempted using hydrogels and polymeric micellar systems which are able to condense DNA by forming a polyplex micelle through polyion complex formation [99,100], similarly to poloxamer PF68 and poloxamine T908 polymeric micelles, alginate-, self-assembling RAD16-I peptides- or polypseudorotaxane gels [101], and PU scaffolds carrying rAAV vectors [102]. Such systems were employed to overexpress TGF-β [103], an interleukin-1(IL-1) receptor antagonist (IL-1Ra), and SOX9 [102,103] as a means to safely target human MSCs (hMSCs) [102,103] and enhance their potential for chondrogenesis and immunomodulation [103], applied to experimental models of cartilage defects in situ for biological joint resurfacing. Moreover, mechanical loading of these structures showed to be an advantageous strategy to promote the formation of an ECM cartilage-like tissue [102].

## 6. Scaffold-Mediated Nonviral In Vitro Gene Delivery

A large range of biomaterial scaffolds have been applied to design nonviral gene delivery systems capable to release, in a sustainable and controlled way, therapeutic genes in desired tissues [76,104] (Table 3) including articular cartilage [105,106] and bone [107,108,109,110,111,112,113,114]. In the field of bone regeneration, which is of high relevance to restore the subchondral bone defect, collagen-based scaffolds have been widely used either alone [107,109,111,112,115] or combined with ceramic particles [109,113,114] to deliver pDNAs encoding for BMP-2 [108,110,112,113,114], BMP-7 [114], TGF-β1 [105,106], PDGF [107,109,111], VEGF [108,109,113], or FGF-2 [112]. Chitosan has been widely used as a scaffolding material in different cartilage tissue engineering approaches due to its cationic nature, acting as healing accelerator and exhibiting antimicrobial activities [116]. Likewise, a supporting role for chitosan in cell proliferation has been also documented [117]. A porous chitosan scaffold loaded with hybrid hyaluronic acid (HA)/chitosan/pDNA nanoparticles encoding TGF-β1 promoted chondrocyte proliferation in vitro [106]. In order to design systems compatible with the structure of osteochondral unit, different composite scaffolds were tested to optimize the regeneration of both cartilage and subchondral bone [93,118,119,120,121]. Yi-Hsuan et al. synthetized a bilayer scaffold composed of type-I collagen and HAp through mineralization of hydroxyapatite nanocrystals. The system was loaded with pDNA-BMP-2 or TGF-β3/calcium phosphate multi-shell nanoparticles conjugated with polyethyleneimine. In vitro assays showed long-term transgene expression, prompting MSCs differentiation into osteogenic and chondrogenic lineages [121]. Recently, a fibrin-based hydrogel activated with an mRNA coding for the transcription factor SOX9 was reported to promote MSC chondrogenesis in vitro, with improved expression of *sox9* in progenitor cells compared with the administration of hydrogels activated with pDNA encoding for this transcription factor [122]. Similarly, micro-macro biphasic calcium phosphate (MBCP) granules activated with an mRNA for BMP-2 were described for their ability to support MSC osteogenic differentiation with triggered higher expression of type-I collagen and osteocalcin relative to the use of activated fibrin hydrogels [123].

## 7. Scaffold-Mediated Nonviral In Vivo Gene Delivery for Osteochondral Repair

Gene delivery from implantable, acellular porous scaffolds represents a versatile approach to promote osteochondral repair by stimulating cell migration and functional tissue development in situ. Biomaterials have been employed to deliver adsorbted nonviral vectors, enhancing their efficacy by delaying degradation and locally maintaining therapeutic concentrations (Table 3). For example, a gene activated matrix (GAM) based on a multi-cistronic plasmid encoding for both BMP-2 and BMP-7 (pDNA-BMP-2/7) complexed with chitosan nanoparticles within a collagen-HA matrix promoted osteogenesis in a critical-size calvarial defect in rats [114]. Four weeks post-implantation in vivo, the designed system induced significantly more bone tissue formation compared with those GAM containing pDNA-BMP-2 alone [114]. Different hydrogels scaffolds based on natural polymers such as alginate [124,125,126,127,128,129,130], carboxymethylcellulose [131], chitosan [132,133,134], gelatin [135], or synthetic ones such as oligo(poly(ethylene glycol) fumarate) (OPF) [136] and polyethylene-glycol (PEG) [137], have been studied as nonviral gene delivery systems for cartilage [118,120], bone [124,125,127,130,131,132,133,134,135,137], or osteochondral repair [93,119,121,126,128,129,136]. Mikos et al showed in a rat osteochondral defect model that implantation of an acellular OPF-based hydrogel loaded in a spatial fashion with DNAs encoding for RUNX2 and the SOX trio complexed with a poly(ethylenimine)-HA (bPEI-HA) delivery vector significantly improved tissue healing relative to empty hydrogels or either factor alone [136].

Another approach uses cellularized scaffolds in the context of nonviral in vivo gene delivery. Constructs based on a gelatin sponge scaffold loaded with MSCs and pullulan-spermine/pDNA complexes encoding for TGF-β1 induced superior cartilage repair compared with controls at 2 months post-implantation in vivo in an osteochondral defect (2 mm in diameter, 3 mm in depth) in rats [105]. Kelly et al developed alginate-based gene activated hydrogels by loading nanohydroxyapatite complexed-BMP-2 and TGF-β3-DNA and MSCs [128]. These systems were able to support transfection of encapsulated MSCs and directed their phenotype toward either a chondrogenic or an osteogenic phenotype in vitro, depending on whether TGF-β3 and BMP-2 were delivered in combination. More recently, 3D-printed pore-forming bioinks that maintain a sustainable transfection by modulating its porosity were synthetized [129]. These gene-activated systems combined alginate-methylcellulose hydrogels loaded with plasmids encoding for osteogenic (BMP-2) or chondrogenic (TGF-β3, BMP-2, SOX9) genes. When implanted subcutaneously in mice in combination with networks of 3D-printed thermoplastic fibers, these 3D constructs supported the development of a vascularized, bony tissue surrounded by a cartilage layer after 4 weeks in such an ectopic location.

After orthotopic implantation of a hybrid scaffold PLGA filled with fibrin gel and loaded with MSCs and pDNA-TGF-β1 complexed with a cationized chitosan derivative in rabbit osteochondral defects, improved cartilage repair was evidenced at 12 weeks compared with control constructs in the absence of pDNA-TGF-β1 or bone marrow-derived MSCs [118]. When the same scaffolds were used to deliver PEO-b-poly(l-lysine) (PEO-b-PLL)/pDNA-TGF-β1 complexes, improved repair of both cartilage and subchondral bone compared with controls was seen after 12 weeks in lapine osteochondral defects [93]. A similar tendency was observed by implantation of a bilayered gene-activated osteochondral scaffold structure with MSCs [119]. The chondrogenic layer consisted of a plasmid TGF-β1-activated chitosan-gelatin scaffold and the osteogenic layer of a plasmid BMP-2-activated HAp/chitosan-gelatin scaffold. When implanted in lapine osteochondral defects, this construct appeared to qualitatively support both articular cartilage and subchondral bone repair after 12 weeks, although no (semi-)quantitative in vivo data were presented [119].

## 8. Scaffold-Mediated Viral In Vitro Gene Delivery for Osteochondral Repair

Many studies immobilize active viral vectors to solid scaffolds, for example by using PLL, creating a biomechanically functional scaffold system (Table 4). Guilak et al generated a self-contained bioactive scaffold capable of mediating stem cell differentiation and formation of a cartilaginous ECM by immobilizing lentiviral vectors on woven PCL scaffolds, an FDA-approved biocompatible aliphatic polyester [138,139]. Such scaffold-mediated gene delivery of TGF-β3 induced robust cartilaginous ECM formation by hMSCs and was as effective as traditional differentiation protocols involving medium supplementation with TGF-β3 protein [140]. A doxycycline-inducible lentiviral vector was capable to transduce MSCs in monolayer or in a 3D arrangement within woven PCL scaffolds to enable a tunable IL-1Ra production as an anti-inflammatory actor. In the presence of IL-1, the IL-1Ra-overexpressing engineered cartilage produced cartilage-specific ECM, while resisting the IL-1-induced upregulation of matrix metalloproteinase (MMP) and, at the same time, maintaining mechanical properties similar to native articular cartilage. In a continuation from this study, the same group engineered functional cartilage anatomically shaped scaffolds capable of inducible and tunable expression of IL-1Ra. Thus, 3D hemispherical scaffolds based on woven PCL fibers were fabricated and seeded with human adipose-derived stromal cells (ASCs). Doxycycline (dox)-inducible lentiviral vectors encoding for eGFP or IL-1Ra transgenes were then immobilized into the PCL scaffolds and constructs were cultured in chondrogenic medium for 28 days. Constructs showed uniform tissue growth and adapted cartilage properties while maintaining their anatomic architecture throughout culture. IL-1Ra-overexpressing constructs produced IL-1Ra (~1 μg/mL) as a result of dox-controlled induction. Likewise, a significant increase of MMP activity was observed in the conditioned media of eGFP-expressing constructs upon IL-1 treatment, but not in IL-1Ra-overexpressing constructs [141]. Chondrogenesis in PCL scaffolds was also induced in MSCs from human bone marrow by rAAV vector gene transfer of SOX9 upon. Prolonged, effective SOX9 expression was reported in the constructs for at least 3 weeks in vitro, leading to enhanced chondrogenic activities by deposition of proteoglycans and increased type-II collagen content in the cells without affecting it proliferative activities. These findings reveal the therapeutic potential of providing rAAV-modified marrow concentrates within 3D-woven PCL scaffolds [142]. Among the large variety of solid scaffolds available for cartilage repair [143], PCL scaffolds present significant advantages as the surface of this low immunogenic, biodegradable compound can be grafted with poly(sodium styrene sulfonate) (pNaSS), a bioactive polymer that facilitates protein adsorption and stimulates reparative cellular responses (adhesion, proliferation) [144]. Overexpression of sox9 in human bone marrow aspirates via rAAV vectors delivered by PCL films functionalized via grafting with pNaSS increased chondrogenic differentiation activities in the aspirates while containing premature osteogenesis and hypertrophy without impacting cell proliferation, with more potent effects noted when using pNaSS-grafted films in vitro [145]. Another study investigated the combined effect of complex mechanical stimulation and adenoviral-mediated overexpression of BMP-2 on hMSC chondrogenesis. Human MSCs transduced with Ad.BMP-2 were encapsulated in a fibrin hydrogel seeded into biodegradable PU scaffolds were stimulated mechanically for 7 or 28 days in chondrogenic medium without growth factors to mimics an in vivo environment, while controls cells were left un-transduced [146]. Transduction with Ad.BMP-2 led to a notable expression of the chondrogenic genes aggrecan and Sox9 upon mechanical stimulation. Besides, the glycosaminoglycans (GAGs)/DNA ratios were reduced following BMP-2 overexpression upon mechanical stimulation [146].

Cartilage-derived matrix (CDM) is another interesting scaffold material due to its chondroinductive capacity and its ability to support endochondral ossification. A recent study aimed to engineer anatomically-shaped cartilage and bone CDM constructs with the ability to inhibit inflammatory processes. Controlled induction of IL-1Ra expression following scaffold-mediated lentiviral gene delivery protected CDM hemispheres from inflammation-mediated degradation, and supported robust bone and cartilage tissue formation even in the presence of IL-1. Moreover, concentric CDM hemispheres resembling the femoral head overexpressing the chondrogenic TGF-β3, or the osteogenic BMP-2 transgenes could be fused into a single bi-layered osteochondral construct [147].

Using hydrogels as biomaterials, controlled delivery via polymeric micelles of rAAV vectors enhanced their temporal and spatial presentation into their targets. Delivering of rAAV vectors via PEO and PPO (poloxamer and poloxamine) polymeric micelles as a means to overexpress TGF-β in human OA chondrocytes resulted in increased proteoglycan deposition and higher cell numbers, thus providing potential tools to remodel human OA cartilage [148]. Another study examined whether a fibrin PU hybrid scaffold provides a favorable environment for the effective chondrogenic differentiation of hMSCs overexpressing SOX9 via rAAV-mediated gene transfer when cultured in rotating bioreactors in vitro. hMSCs could be modified via rAAV to overexpress SOX9 over an extended period within these scaffolds, leading to an improved cell chondrogenic differentiation in such a hydrodynamic environment relative to control (reporter *lacZ*) vector treatment [112].

## 9. Scaffold-Mediated Viral In Vivo Gene Delivery for Osteochondral Repair

Although several groups have applied transduced cells for musculoskeletal repair in solid [54,69,149,150] or hydrogel scaffolds for cartilage [59] and bone repair [149], comparably few have used viral vectors immobilized to scaffolds without ex vivo transduced cells for in vivo applications (Table 4). So far, only one published study performed a biomaterial-guided in vivo delivery of a gene vector in an orthotopic large animal model of osteochondral repair. In this study, a thermosensitive hydrogel based on PEO-PPO-PEO poloxamers, capable of controlled release of a rAAV vector overexpressing SOX9 was applied to a full-thickness chondral defect treated with microfracture in a minipig model. PEO-PPO-PEO (PF127) hydrogels carrying either the candidate rAAV-FLAG-h*sox9* vector (*sox9*/hydrogel) or a control rAAV-*lacZ* vector (*lacZ*/hydrogel) were directly applied into defects treated with microfracture. Four weeks postoperatively, the individual histological scoring parameters “integration”, “cellular morphology”, “matrix staining” and the total cartilage repair score were significantly improved following the *sox9*/hydrogel application relative to all other groups, together with an increased deposition of type-II collagen in the *sox9*/hydrogel *versus lacZ*/hydrogel defects or when applying the *lacZ* vector in a hydrogel-free form. Next, the apparent absence of an immune response in all defects (lack of expression of CD3 for T-lymphocytes, of CD11b for activated macrophages, and of human leukocyte antigen isotype DR alpha—HLA-DRα—for class II major histocompatibility complex—MHC—antigens) supported the use of such PEO-PPO-PEO poloxamers to protect rAAV-mediated gene transfer from neutralization by antibodies directed against the AAV capsid. Although not directly applied to an osteochondral defect model, this study showed by a comprehensive analyses of the entire osteochondral unit that rAAV-FLAG-h*sox9*/PEO-PPO-PEO hydrogel-augmented microfracture significantly improves osteochondral repair [151].

## 10. Clinical Scaffolds for Osteochondral Repair

No scaffold is currently in routine clinical use that is capable of delivering a gene vector to sites of osteochondral damage. Also, entry in clinical trials to treat osteochondral defects has been granted to only a few scaffolds so far, among which are a nanocomposite three-layered collagen-HAp scaffold, a PLGA-calcium-sulfate bilayer scaffold, and an aragonite-based scaffold [152]. Clinical results were either not satisfying, or limited to a few reported case series, necessitating more high-level studies with longer follow-up [152].

In contrast, a variety of scaffolds are in clinical development to deliver articular chondrocytes in the context of ACI. These scaffolds can also be used either with or without cells to cover an osteochondral defect when the bony part of the defect is filled with a bone graft or bone substitutes. Classically, solid scaffolds are used in such clinical situations. They are composed of materials such as type-I/III collagen (MACI™, Novocart 3D™), HA (Hyalograft^®^ C) and PGA, polylactic acid (PLA), and polydioxanone (BioSeed C™). More recently, hydrogels have emerged as a viable alternative, among them type-I collagen (atelocollagen) hydrogels (Koken Atelocollagen Implant), HA (CARTISTEM™) hydrogels, albumin and HA hydrogels (Novocart Inject™), fibrin (Chondron™), and agarose and alginate hydrogels (Cartipatch™). Currently, not all commercial products are available for clinical use. While these scaffolds have been used largely to deliver articular chondrocytes, they may also be used alone as a cell-free approach.

## 11. Conclusions

A variety of biomaterials have been employed as nonviral or viral gene carriers for steochondral repair in vitro, including hydrogels, solid scaffolds and hybrid scaffolds, supporting the concept of advanced biomaterial-guided delivery of gene carriers as an attractive therapeutic option for osteochondral repair in vivo. Such biomaterial-mediated gene therapy provides both a template for endogenous cell migration, infiltration and tissue formation while simultaneously promoting overexpression of therapeutic proteins in a sustained and locally determined fashion [76]. As demonstrated, a site-specific delivery of inducible transgenes confers spatial and temporal control over both scaffold remodeling and osteochondral neotissue composition [147]. Of note, a combined gene delivery approach may also provide immunomodulatory properties that allow for chondrogenesis in the presence of pathologic levels of degenerative factors among which IL-1, a critical step that may enhance the long-term success of osteochondral repair in the case of injuries or the presence of OA [153]. Since the techniques of scaffold design are highly sophisticated, such scaffold-mediated gene delivery may be potentially used to generate both large anatomically shaped but also cartilage constructs individualized to the 3D defect morphology while possessing the capability for a controlled gene delivery [141]. Moreover, the PEO-PPO-PEO copolymers controlling the release of the gene vectors are promising biomaterials for in vivo rAAV delivery, supporting repair in conditions where protection against potentially damaging host immune responses may be needed.

From a clinical point of view, the proven capability to deliver thermosensitive hydrogels that display a sol-gel transition at body temperature while simultaneously controlling the release of therapeutic gene vectors conceptually supports minimally invasive in vivo application strategies, an attractive feature for osteochondral defects that are located in joints that are more difficult to access via an arthrotomy, for example, the hip joint. These current advances support the concept of a scaffold-mediated gene delivery for osteochondral repair in the future.

## Figures and Tables

**Table 1:** Gene transfer vectors.

Systems	Vectors	Efficacy	Integration	Features
Nonviral	naked pDNA	very low	no	very short-term expression, very low efficiency
	lipoplexes	low	no	short-term expression, low immunogenicity, cytotoxicity at high concentrations
	polyplexes	low	no	short-term expression, low immunogenicity, cytotoxicity at high concentrations
	lipopolyplexes	medium	no	short-term expression, low immunogenicity, low cytotoxicity
	nanoparticles	medium	no	short-term expression, costly, quality control difficulties
	transposons	medium	yes	long-term expression, low immunogenicity, low cytotoxicity
Viral	adenoviral	very high	no	short-term expression, strong immunogenicity
	retroviral	high	yes	long-term expression, strong immunogenicity
	baculoviral	high	no	short-term expression
	rAAV	very high	no	long-term expression, low immunogenicity

Abbreviations: pDNA: plasmid DNA; rAAV: recombinant adeno-associated viral vector.

**Table 2:** Properties of scaffolds used for osteochondral repair.

Systems	Biocompatibility	Biodegradation	Mechanical/ Physico-Chemical Properties	Biological Properties
hydrogels (alginate, chitosan, collagen, gelatin, etc.)	high	high	poor mechanical strength, high porosity and swelling ratio	ECM-like properties
solid scaffolds (PCL, PLGA, etc.)	low	low	high mechanical strength, tuneable properties	controlled release of biomolecule cargos
hybrid scaffolds (fibrin/PLGA, gelatin/collagen, etc.)	moderate-high	moderate	combination of hydrogels and solid scaffolds properties	high cell adhesion and sustained release profiles

Abbreviations: PCL: poly(ε-caprolactone); PLGA: poly(lactic-co-glycolic acid); ECM: extracellular matrix.

**Table 3:** Scaffold-mediated nonviral gene delivery.

Vectors	Genes	Scaffolds	In Vitro Target Cells	In Vivo Models	Applications	Ref.
PEI complexes	PDGF	collagen	BMSCs	rat	bone repair (cell proliferation, osteogenesis)	[107]
	VEGF, BMP-2	collagen-nHA	rMSCs	rat	bone repair (cell proliferation, osteogenesis, angiogenesis)	[108]
	VEGF, PDGF	collagen	BMSCs	rat	bone repair (cell proliferation, osteogenesis, angiogenesis)	[109]
	PDGF	collagen	hPLFs hGFs	rat	bone repair (cell proliferation, osteogenesis)	[111]
	FGF-2 BMP-2	collagen	BMSCs	rabbit	bone repair (cell proliferation, osteogenesis, angiogenesis)	[112]
	GFP, luc	collagen-nHA	rMSCs	-	transgene expression	[115]
	OSX	CMC nanogel	hMSCs	-	bone repair (osteogenesis)	[131]
bPEI-HA complexes	SOX trio, RUNX2	OPF hydrogel	-	rat	osteochondral repair (osteo-/chondrogenesis)	[136]
CaP/PEI nanoparticles	TGF-β3, BMP-2	collagen-nHA	hMSCs	-	osteochondral repair (osteo- /chondrogenesis)	[121]
CaP nanoparticles	BMP-2	3D-printed alginate hydrogel	gMSCs	mouse	bone repair (osteogenesis)	[130]
	BMP-2	alginate hydrogels	MC3T3-E1	mouse	bone repair (osteogenesis)	[124]
nHA particles	TGF-β3, BMP-2, SOX9	3D-printed alginate-MC hydrogel	hMSCs	mouse	osteochondral repair (osteo-/chondrogenesis)	[129]
	TGF-β3, BMP-2	alginate hydrogels	MSCs	-	bone repair (osteogenesis)	[128]
chitosan nanoparticles	VEGF, BMP-2	collagen-nHA	rMSCs	rat	bone repair (cell proliferation, osteogenesis, angiogenesis)	[113]
	BMP-2, BMP-7	collagen-nHA	rMSCs	rat	bone repair (osteogenesis)	[114]
	ASO, TNF-α	gelatin-chitosan hydrogel	RAW 264.7	mouse	bone repair (suppression of osteoclastogenesis)	[132]
	BMP-2	chitosan hydrogel	-	rat, beagle dog	bone repair (osteogenesis)	[134]
	BMP-2	chitosan hydrogel	hPDLCs	-	bone repair (osteogenesis)	[133]
hyaluronic acid-chitosan nanoparticles	TGF-β1	porous chitosan	chondrocytes	-	cartilage repair (chondrogenesis)	[106]
PEO-b-PLL complexes	TGF-β1	PLGA	rbMSCs	rabbit	osteochondral repair (chondrogenesis)	[93]
pullulan-spermine complexes	TGF-β1	gelatin sponge	rMSCs	rat	cartilage repair (chondrogenesis)	[105]
TMC complexes	TGF-β1	PLGA sponge	BMSCs	rabbit	cartilage repair (chondrogenesis)	[119]
superFect complexes	BMP-2	PLGA	skull-derived osteoblasts	mouse	bone repair (osteogenesis)	[110]
	BMP-2	PEG hydrogel	hFOB	pig	bone repair (osteogenesis)	[137]
lipofectamine complexes	TGF-β1	PLGA/fibrin hydrogel	rMSCs	rabbit	cartilage repair (chondrogenesis)	[120]
FuGene6 complexes	hIGF-I	calcium alginate hydrogel	BMSCs	goat	osteochondral repair (osteo-/chondrogenesis)	[126]
naked pDNA	TGF-β1, BMP-2	CG/HCG	rMSCs	rabbit	osteochondral repair (osteo-/chondrogenesis)	[119]
	BMP-2	alginate hydrogel	hMSCs, MG-63	mouse	bone repair (osteogenesis)	[125]
	BMP-2	alginate hydrogel	gMSCs	goat	bone repair (osteogenesis)	[127]
	BMP-2	collagen and gelatin hydrogels	-	mouse	bone repair (osteogenesis)	[135]
mRNA 3DfectIN^®^ complexes	SOX9	fibrin hydrogel	hMSCs	-	cartilage repair (chondrogenesis)	[122]
mRNA DreamFect Gold complexes		fibrin gel or MBCP granules	rMSCs	-	bone repair (osteogenesis)	[123]

Abbreviations: PEI: polyethylenimine; PDGF: platelet derived growth factor; BMSCs: bone marrow-derived stromal cells; VEGF: vascular endothelial growth factor; BMP-2: bone morphogenetic protein 2; rMSCs: rat mesenchymal stromal cells; hPLFs: human periodontal ligament fibroblasts; hGFs: human gingival fibroblasts; FGF-2: basic fibroblast growth factor; GFP: green fluorescent protein; *luc*: Firefly luciferase; nHA: nanohydroxyapatite; OSX: osterix; CMC: carboxymethylcellulose; hMSCs: human mesenchymal stromal cells; bPEI: branched polyethylenimine; HA: hyaluronic acid; SOX trio: sex-determining region Y-type high mobility group box 5, 6, and 9; RUNX2: runt-related transcription factor 2; OPF: oligo polyethylene glycol fumarate; CaP: calcium phosphate; TGF-β3: transforming growth factor beta 3; gMSCs: goat mesenchymal stromal cells; MC3T3-E1: preosteoblasts cell line; SOX9: sex-determining region Y-type high mobility group box 9; MC: methylcellulose; BMP-7: bone morphogenetic protein 7; ASO: antisense oligonucleotide; RAW 264.7: macrophage cell line; hPDLCs: human periodontal ligament cells; TGF-β1: transforming growth factor beta 1; PEO-b-PLL: poly(ethylene oxide)-b-poly(l-lysine); PLGA: poly(lactic-co-glycolic acid); rbMSCs: rabbit mesenchymal stromal cells; TMC: *N*,*N*,*N*-trimethyl chitosan chloride; PEG: polyethylene glicol; hFOB: human fetal osteoblasts; hIGF-I: insulin-like growth factor I; CG: chitosan-gelatin; HCG: hydroxyapatite/chitosan-gelatin; pDNA: plasmid DNA; mRNA: messenger RNA; MG-63: homo sapiens bone osteosarcoma cell line; MBCP: micro-macro biphasic calcium phosphate.

**Table 4:** Scaffold-mediated viral gene delivery.

Vectors	Genes	Scaffolds	In Vitro Target Cells	In Vivo Models	Applications	Ref.
lentiviral	IL-1Ra	PCL	ASCs	-	cartilage repair (reduction of MMP activity)	[141]
	eGFP, TGF-β3, BMP-2, IL-1Ra	CDM	hMSCS	-	cartilage repair (protection against tissue degradation)	[147]
rAAV	SOX9	PU	hMSCs	-	cartilage repair (cell proliferation, ECM deposition, reduced hypertrophy)	[102]
	SOX9	PCL	hBMA	-	cartilage repair (cell proliferation, ECM deposition, reduced hypertrophy)	[142]
	SOX9	pNaSS-grafted PCL	hBMA		cartilage repair (cell proliferation, ECM deposition)	[145]
	TGF-β1	PEO-PPO-PEO micelles	chondrocytes	-	cartilage repair (cell proliferation, ECM deposition)	[148]
	SOX9	PEO-PPO-PEO hydrogel	-	minipig	osteochondral repair (ECM deposition)	[151]
adenoviral	BMP-2	PU	hMSCS		cartilage repair (ECM deposition)	[146]
	BMP-2, TGF-β3	DBM	BMSCs	pig	cartilage repair (ECM deposition)	[149]
	TGF-β1	PGA	BMSCs	mice	cartilage repair (ECM deposition)	[54]
	SOX9	PGA	BMSCs	rabbit	cartilage repair (ECM deposition)	[150]
baculoviral	TGF-β1, BMP-6	PLGA	rASCs	rabbit	cartilage repair (neocartilage formation)	[69]

Abbreviations: IL-1Ra: interleukin-1 receptor antagonist; PCL: poly(ε-caprolactone); ASCs: adipose-derived stem cells; eGFP: enhanced green fluorescent protein; TGF-β3: transforming growth factor beta 3; BMP-2: bone morphogenic protein 2; CDM: cartilage-derived matrix; hMSCs: human mesenchymal stromal cells; SOX9: sex-determining region Y-type high mobility group box 9; PU: polyurethane; hBMA: human bone marrow aspirate; pNaSS: poly(sodium styrene sulfonate); TGF-β1: transforming growth factor beta 1; PEO-PPO-PEO: poly(ethylene oxide)-poly(propylene oxide)-poly(ethylene oxide); DBM: demineralized bone matrix; BMSCs: bone marrow-derived stromal cells; PGA: polyglycolic acid; BMP-6: bone morphogenic protein 6; PLGA: poly(lactide-co-glycolide); rASCs: rabbit adipose-derived stem cells; MMP: matrix metalloproteinases.
